# Cellular dynamics of neuronal migration in the hippocampus

**DOI:** 10.3389/fnins.2015.00135

**Published:** 2015-04-24

**Authors:** Kanehiro Hayashi, Ken-ichiro Kubo, Ayako Kitazawa, Kazunori Nakajima

**Affiliations:** Department of Anatomy, Keio University School of MedicineTokyo, Japan

**Keywords:** hippocampus, migration, climbing mode, Ammon's horn, dentate gyrus, layer pattern

## Abstract

A fine structure of the hippocampus is required for proper functions, and disruption of this formation by neuronal migration defects during development may play a role in some psychiatric illnesses. During hippocampal development in rodents, pyramidal neurons in the Ammon's horn are mostly generated in the ventricular zone (VZ), spent as multipolar cells just above the VZ, and then migrate radially toward the pial surface, ultimately settling into the hippocampal plate. Although this process is similar to that of neocortical projection neurons, these are not identical. In addition to numerous histological studies, the development of novel techniques gives a clear picture of the cellular dynamics of hippocampal neurons, as well as neocortical neurons. In this article, we provide an overview of the cellular mechanisms of rodent hippocampal neuronal migration including those of dentate granule cells, especially focusing on the differences of migration modes between hippocampal neurons and neocortical neurons. The unique migration mode of hippocampal pyramidal neurons might enable clonally related cells in the Ammon's horn to distribute in a horizontal fashion.

## Introduction

The hippocampal formation is a unique structure comprising the Ammon's horn (the hippocampus proper), dentate gyrus, entorhinal cortex, parasubiculum, presubiculum, and subicular complex. In the rodent brain, this architecture is located on and around the convexly curved medial lobule of the lateral cortex and is dorsally continuous with the neocortex. The hippocampus is a part of the limbic circuit and is functionally associated with spatial learning, as well as short- and long-term memory. In addition, functional magnetic resonance imaging (fMRI) analyses of some neuropsychiatric disorders have indicated its involvement in various types of mental activities; for example, decreased hippocampal volume was reported in patients with depression or post-traumatic stress disorder (PTSD) (Campbell et al., 2004; Woon et al., 2010). Anatomical abnormalities in the hippocampus are also observed in pathological conditions of some neuropsychiatric disorders, such as epilepsy, lissencephaly, and schizophrenia (Baulac et al., 1998; Harrison, 2004; Donmez et al., 2009). Some of these symptoms are thought to be associated with the migration deficit of hippocampal neurons during development (Barkovich et al., 1991; Dobyns et al., 1996; Montenegro et al., 2006). Neuronal migration in the neocortex is well-studied, and the cellular dynamics and molecular mechanisms involved in neuronal migration are also well-understood. Because the hippocampus and neocortex are included in the cerebral cortex, their neuronal migration was thought to be similar. However, differences in neuronal migratory behavior between these regions exist. Studies on cellular behavior of hippocampal neurons are broadly classified into two categories in terms of their methods, classical cellular labeling and molecular biological approaches. Classical techniques, such as Golgi staining and [3H] thymidine autoradiography labeling, were used to discover neuronal origins, cellular arrangements, neuronal migration paths, and neuronal morphologies in the hippocampus (Bayer, 1980; Nowakowski and Rakic, 1981; Rakic and Nowakowski, 1981; Altman and Bayer, 1990a,b,c). Furthermore, the development of molecular biological approaches, such as *in utero* electroporation, *in utero* virus transfer, and generation of transgenic mice, has shed light on the cellular dynamics of migrating neurons, successive behavior of neurons, neuronal lineages, and molecular mechanisms of hippocampal development (Nakahira and Yuasa, 2005; Li et al., 2009; Kitazawa et al., 2014; Seki et al., 2014; Xu et al., 2014).

In this review, we describe cellular dynamics and molecular mechanisms of migration of pyramidal neurons in the Ammon's horn and granule cells in the dentate gyrus during hippocampal development. There are three distinct hippocampal neuroepitheliums—the Ammonic neuroepithelium, the primary dentate neuroepithelium, and the fimbrial glioepithelium (Altman and Bayer, 1990a). Pyramidal neurons in the Ammon's horn are mainly generated from the Ammonic neuroepithelium and undergo radial migration to reach their final destination (Altman and Bayer, 1990b; Nakahira and Yuasa, 2005; Kitazawa et al., 2014), whereas cells comprising the dentate gyrus are originally produced from the primary dentate neuroepithelium (dentate notch), move in a migratory stream, and then migrate radially to form the dentate granule cell layer (Altman and Bayer, 1990a,c; Nakahira and Yuasa, 2005; Li et al., 2009; Seki, 2011; Li and Pleasure, 2014; Seki et al., 2014). We also compare neuronal migration between the neocortex and the hippocampus proper during development.

## Migration of neocortical pyramidal neurons

**Figure 1:**
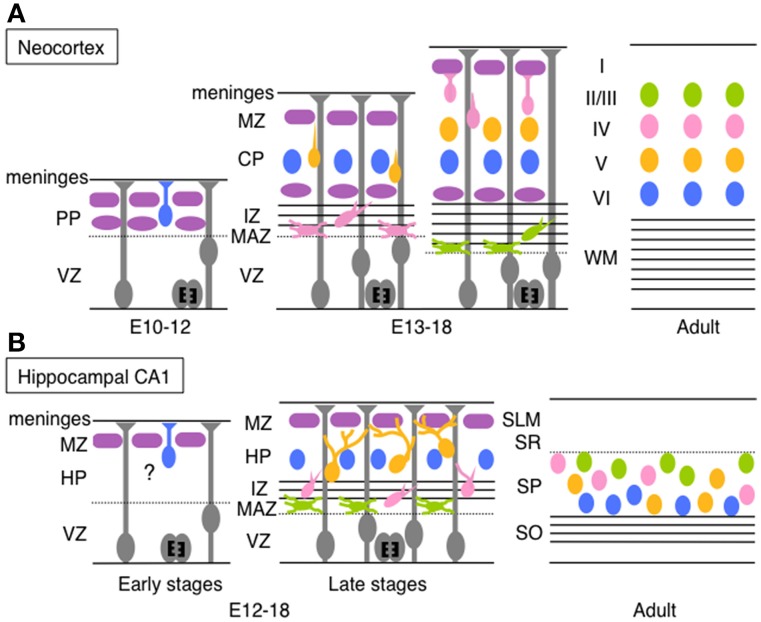
**Schematic diagrams of migration and layer arrangement on the neocortex and hippocampal CA1 during cortical development. (A)** Neocortical neurons born between E10 and E12 radially migrate using the somal translocation mode. In contrast, late-born neurons transform their migration mode sequentially to multipolar migration, locomotion mode, and terminal translocation mode during their radial migration. These neurons form neocortical layers in a birthdate-dependent inside-out manner. **(B)** Hippocampal CA1 neurons born at late developmental stages change the migration mode to multipolar migration and then to the climbing mode. The migration mode used by early-born CA1 neurons remains unknown (somal translocation mode is a candidate). The layer arrangement in the Ammon's horn is thought to occur roughly in a birth-date dependent inside-out manner (another claim was also reported; see text for details). PP, preplate; VZ, ventricular zone; MZ, marginal zone; CP, cortical plate; IZ, intermediated zone; MAZ, multipolar cell accumulation zone; WM, white matter; HP, hippocampal plate; SLM, stratum lacunosum-moleculare; SR, stratum radiatum; SP, stratum pyramidale; SO, stratum oriens.

Before describing migration of hippocampal neurons, we briefly outline migration of pyramidal neurons during rodent neocortical development (Figure 1A) to compare the migration mode between hippocampal pyramidal neurons and neocortical neurons (for a detailed illustration of neocortical neuronal migration, see reviews by Tabata et al., 2012; Evsyukova et al., 2013; Tan and Shi, 2013; Sekine et al., 2014). Pyramidal neurons generated in the neocortical ventricular zone (VZ) undergo morphological transformation before migrating up beneath the marginal zone (MZ), as summarized below.

Neocortical pyramidal neurons are generated from radial glial cells in the VZ (Miyata et al., 2001; Noctor et al., 2001) or from basal progenitors or basal radial glia in or around the subventricular zone (SVZ) (Noctor et al., 2004; Shitamukai et al., 2011; Wang et al., 2011). Neurons produced in the VZ remain there for at least 10 h with an apical process reaching the ventricular surface. The cells then move to just above the VZ (multipolar cell accumulation zone, MAZ), where they assume multipolar morphology and stay for about 1 day (Tabata and Nakajima, 2003; Tabata et al., 2009). Multipolar neurons in the MAZ repeatedly extend and retract multiple thin processes, and slowly wander and move toward the cortical plate (CP) (Tabata and Nakajima, 2003; Tabata et al., 2009). This unique behavior of multipolar neurons is called “multipolar migration.” The multipolar neurons then transform into bipolar cells with a leading process extending from a spindle-shaped cell body. These bipolar neurons migrate radially through the intermediated zone (IZ) and the CP along with a radial glial fiber. This migration mode is called “locomotion” (Rakic, 1972; Nadarajah et al., 2001). When the leading process of migratory neurons reaches the MZ, the neurons are thought to anchor the tip of the leading process in the MZ and leave from the radial glial fiber. Then, the neuronal cell body is pulled up while shortening the leading process and the cells stop just beneath the MZ (Nadarajah et al., 2001; Sekine et al., 2011). This final migration mode is termed “terminal translocation.” Neurons born in the mouse VZ at E14, for example, take about 4–5 days to complete their migration (Ajioka and Nakajima, 2005). Because newly generated pyramidal neurons pass through earlier-born neurons before reaching beneath the MZ, pyramidal neurons are arranged in a birth-date-dependent inside-out manner, in which earlier-born neurons are positioned in the deep layers and later-born neurons are located in the more superficial layers in the CP (Angevine and Sidman, 1961).

## Migration of hippocampal CA1 pyramidal neurons

The Ammon's horn is compartmentalized into the CA1, CA2, and CA3 along with the transverse axis, and horizontally divided into several ordered layers—stratum oriens, stratum pyramidale (SP or the pyramidal cell layer), stratum radiatum (SR), and stratum lacunosum-moleculare (in only the CA3 region, the stratum lucidum exists between the SP and the SR). Pyramidal neurons in the hippocampal CA1 region are generated in the Ammonic neuroepithelium mainly from E16 to E20 with the peak around E18–19 in rats (Bayer, 1980; Altman and Bayer, 1990a,b), and from E12 to E18 with the peak between E14 and E16 in mice (Angevine, 1965; Caviness and Sidman, 1973; Smart, 1982; Kitazawa et al., 2014). Because the generation of pyramidal neurons and neuronal behaviors during development are different between the hippocampal CA1 and the CA3, we describe migratory behaviors of CA1 pyramidal neurons first and then those of CA3 neurons later (details of CA2 pyramidal neuronal migration are not well-known). Pyramidal neurons in the hippocampal CA1 region are mostly generated in the VZ, while a small population is produced from basal progenitors in the SVZ (Kitazawa et al., 2014). The newly born neurons leave the VZ and stay just above the VZ. These post-mitotic cells transform into multipolar cells with multiple thin processes and slowly move toward the IZ with repeated extension and retraction of these processes (Nakahira and Yuasa, 2005; Kitazawa et al., 2014). Existence of these multipolar neurons in the Ammon's horn has also been identified in the rabbit (Stensaas, 1967a,b) and the monkey (Nowakowski and Rakic, 1979). The time length of assuming multipolar morphology above the VZ (or in the hippocampal MAZ) differs depending on birthdates; neurons born at E12 or E13 stay as multipolar cells within 1 day, whereas those generated at E15 or E16 densely accumulate in the MAZ as multipolar cells for 3–4 days (Kitazawa et al., 2014). When late-born CA1 pyramidal neurons (generated from E14 to E16) move into the IZ, they transform into a bipolar spindle-shaped morphology, with one major leading process and multiple thin processes extending toward various directions (Nakahira and Yuasa, 2005; Kitazawa et al., 2014). These spindle-shaped neurons migrate through the IZ toward the hippocampal plate (HP, future stratum pyramidale), but sometimes transform their morphology back to a multipolar morphology. Upon observation of fixed tissue sections, the neurons seem to migrate along with radial glial fibers in the IZ, even when the fibers bend and curve (Nakahira and Yuasa, 2005). Recent time-lapse imaging has revealed that neurons in the IZ move obliquely at first and gradually migrate radially (Kitazawa et al., 2014), which coincides with the track of radial fibers. Nowakowski and Rakic also identified the apposition of neurons with radial glial fibers in the IZ of the monkey hippocampus in electron microscopic analyses (Nowakowski and Rakic, 1979). Just before neurons enter the HP, they extend one or two major branched leading process(es) with multiple thin processes (Nowakowski and Rakic, 1979; Kitazawa et al., 2014) and touch multiple radial glial fibers at the tip or the middle of the branched processes (Kitazawa et al., 2014). When they migrate through the HP, they dynamically extend and retract branched leading processes as if they were searching for the radial glial fibers. On the other hand, the cell soma of the migrating neuron moves up to the first branching point of the leading process. Subsequently, one of the branches grows and becomes a new leading process, followed by the movement of the cell soma again to the first branching point of the new leading process. In the HP of the CA1, migratory neurons repeat this process, thereby changing their migration scaffold (radial glial fiber) one after another until they reach the top of the HP. Consequently, hippocampal pyramidal neurons move in a zigzag manner, in contrast to the almost straight path of neocortical migrating neurons. Because this hippocampal migration mode is different from the well-known modes of migration, it was termed a “climbing mode” (Kitazawa et al., 2014) (Figure 1B).

## The difference between migration of hippocampal CA1 pyramidal neurons and that of neocortical neurons

There are a couple of similarities in the mode of neuronal migration between the hippocampal CA1 and the neocortex. One is the place of neuronal production, which is located in the VZ and SVZ in both structures. Neurons are generated near the ventricle and principally migrate toward the pial surface. The second similarity is the transformation into multipolar morphology when neurons migrate out of the VZ. The neurons reside in the MAZ for some time, and then transform into a bipolar morphology. However, there are also several major differences between the regions, such as migration mode, length of time required for completion of migration, and alignment pattern of the pyramidal neurons.

During development, neocortical neurons migrate in locomotion and terminal translocation modes through the CP (Nadarajah et al., 2001; Sekine et al., 2011; Evsyukova et al., 2013), while pyramidal neurons in the hippocampal CA1 region adopt a climbing migration mode, at least during the late stages of hippocampal development. Neocortical neurons in the locomotion mode migrate almost straight along individual radial glial fibers in the CP. In the outermost region of the CP [primitive cortical zone, PCZ (Sekine et al., 2011)], they take the terminal translocation mode before stopping beneath the MZ. In contrast, hippocampal neurons in the climbing migration mode migrate in a zigzag manner using several scaffold radial glial fibers in the HP (Kitazawa et al., 2014). The migratory speed for each migration mode is also different. The average migrating speeds of hippocampal CA1 neurons in the climbing mode and neocortical neurons in the locomotion mode are 7.1 and 20.5 μm/h, respectively (Kitazawa et al., 2014). The speed of neocortical neurons in the terminal translocation mode is much faster, up to 50 μm/1–2 h (Sekine et al., 2011). Neocortical migrating neurons in a locomotion mode basically use a single radial glial fiber as the scaffold, whereas hippocampal neurons proceed using multiple radial glial fibers. The difference in these processes may bring about the difference in migration speed. Because cell density in the HP in late developmental stages is much greater than in the CP, with exception of the PCZ, it may be difficult for hippocampal CA1 neurons to migrate straight, unlike locomoting neurons in the neocortical CP. Because the Ammon's horn is widely extended during development (Altman and Bayer, 1990b), it is thought that pyramidal neurons born near the ventricle may need to move obliquely using the climbing mode of migration to fill up layers without gaps.

The second difference is the time spent migrating. Hippocampal CA1 pyramidal neurons generated at E15 or E16 spend 7–9 days to reach their final destinations (Tomita et al., 2011; Kitazawa et al., 2014), whereas it takes only 4–5 days for the neocortical late-born neurons to migrate beneath the MZ, although the migratory distance is much longer in the neocortex. This difference is observed not only in mice, but also in rats (Altman and Bayer, 1990b) and monkeys (Nowakowski and Rakic, 1981). Two factors apparently cause this difference. One is the difference in neuronal migration speed as mentioned above. The other is the time period when neurons remain in the MAZ as multipolar cells, termed “sojourning” cells by Altman and Bayer (1990b). Neocortical neurons spend about 1 day in the MAZ (Tabata et al., 2009). In contrast, the hippocampal CA1 neurons generated in late embryonic days spend almost 3 days in the MAZ, while this period varies depending on their birthdates (Nakahira and Yuasa, 2005; Kitazawa et al., 2014). Why do hippocampal neurons generated during late stages stay in the MAZ so long? There are at least two possibilities. One is the existence of alveolar channels, which are cell-free transient extracellular matrices dispersed in the IZ, just above the MAZ (Altman and Bayer, 1990b). Altman and Bayer displayed that this matrix becomes filled with axonal fibers of an unknown origin, and suggested that multipolar cells in the CA1 region might wait for the appearance of this matrix because of the connection to these axons (Altman and Bayer, 1990b). In addition, Deguchi et al. exhibited that neurons born with the same birthdate in the CA1, CA3, and dentate gyrus had similar gene expression patterns and preferentially connected with each other (Deguchi et al., 2011). Multipolar cells in the CA1 region may leave from the MAZ after CA3 neurons extend their axons and connect with them. In contrast, we showed that axon bundles appeared just above the MAZ at E15 and E16. These axon bundles originated from earlier-born neurons in the hippocampal CA1 region, and when they were transfected with GFP at E13.5, for example, the labeled axon bundles were located just above the multipolar cells at E18.5 (Kitazawa et al., 2014). The appearance of these axonal bundles coincided with the accumulation of multipolar cells in the hippocampal MAZ. CA1 neurons born on earlier days spend a much shorter time in a multipolar morphology and reach the pial surface in a short time, whereas axon bundles are not observed above multipolar cells in these earlier days (Kitazawa et al., 2014). Axonal bundles from earlier-born neurons may interfere with the migration of late-born pyramidal neurons. Future studies are needed to better understand the behavior of multipolar cells.

Finally, the pattern of neuronal alignment in the hippocampus may be different from the neocortex. Labeling experiments using [3H] thymidine indicate that hippocampal laminar formation occurs in a birthdate-dependent inside-out pattern in which earlier-born neurons comprise the deep SP region and later-born neurons join the superficial region, which is similar to the neocortical layer formation (Bayer, 1980; Rakic and Nowakowski, 1981; Altman and Bayer, 1990b). Recently, Xu et al. reported that hippocampal clonally related neurons in the CA1 region are distributed in a horizontal manner, not in a vertical column as neocortical neurons, shown using retroviral labeling and transgenic mice to label clonally related cells (Xu et al., 2014). The authors claimed that hippocampal layer formation did not occur in a birthdate-dependent inside-out pattern, contrary to previous reports. In addition to differences in their analytical method, this disparity might be explained as follows. Because neurons born on a certain day are distributed rather widely in the SP with a birthdate-dependent peak position, neurons with different birthdates are mixed with each other, obscuring the inside-out pattern. In addition to the birthdate-dependent positioning in the vertical/radial axis, however, it is also reasonable that the clonally related neurons are horizontally aligned. Because the SP (or HP) in the Ammon's horn region is horizontally expanded during development and is not as thick as the CP in the neocortex, this suggests the difficulty of clonally related neurons in the hippocampus proper to be aligned in a vertical manner like neocortical clonal neurons. Even if neurons in the HP of the Ammon's horn tend to align in an inside-out pattern, they would also move to cover spaces in the layer yielded by the structural expansion, resulting in lateral/horizontal expansion of clonally related neurons. The climbing mode of migration is thought to be suitable to fill up gaps in the layer, because neurons in this mode can move to various directions, enabling clonal sister neurons to distribute broadly within the layer. Bending of radial glial fibers near the HP/SP would also be partly related to horizontal distribution of sister neurons (Xu et al., 2014), but similar bending of radial glial fibers beneath the CP/subplate is also observed in the neocortex, especially in the lateral part (Tabata and Nakajima, 2001), indicating that the bending morphology of radial fibers cannot fully explain the horizontal distribution of sister neurons in the hippocampus. Schematic models of neuronal behaviors in the neocortex and the Ammon's horn during development are illustrated in Figure 1.

## Migration of hippocampal CA3 pyramidal neurons

The SP in the CA3 region has a unique U-shaped curve that reaches the dentate hilus. In rats, the HP appears from E18 and expands curvilinearly until E21, at which time the layer begins to medially expand and forms a U-shaped structure by E22 (Altman and Bayer, 1990b). The CA3 pyramidal neurons have a neurogenic gradient such that pyramidal neurons near the CA1 region are generated earlier than those near the dentate gyrus (Bayer, 1980). The neurogenesis gradient from ventral to dorsal is also observed in the CA3 region. The neurogenesis of CA3 pyramidal neurons takes place in the VZ between E16 and E20, with a peak between E17 and E18 in rats, which is earlier than the generation of hippocampal CA1 neurons that peak around E18 and E19 in rats (Bayer, 1980; Altman and Bayer, 1990a,b). The generated neurons move to just above the VZ and transform into a multipolar morphology, similar to CA1 pyramidal neurons (Nakahira and Yuasa, 2005). However, CA3 neurons remain longer in the MAZ than CA1 neurons. In mice, neurons generated in the CA3 region at E14 exhibit multipolar morphology even at E18, while CA1 pyramidal neurons born at the same time have already migrated into the HP (Nakahira and Yuasa, 2005). This phenomenon is also observed in rats using administration of [3H] thymidine (Altman and Bayer, 1990b). Reports have shown that the sojourning period for CA3 pyramidal neurons assuming a multipolar morphology is 1 day longer than for CA1 neurons (Altman and Bayer, 1990b; Nakahira and Yuasa, 2005). Altman and Bayer hypothesized that multipolar cells in the CA3 region might wait for a connection with granule cells of the dentate gyrus, resulting in a longer sojourning time (Altman and Bayer, 1990b). Again, the report by Deguchi et al. showed that neurons generated at the same time in different sub-regions connect preferentially with each other (Deguchi et al., 2011), which may also explain a longer sojourn to wait for a connection with dentate gyrus cells. The CA3 pyramidal neurons accumulate in the MAZ for 4 days then migrate upward. The migration modes of CA3 pyramidal neurons are thought to be similar to CA1 pyramidal neurons, though only a limited number of studies have been reported on the behavior of hippocampal CA3 pyramidal neurons (Nakahira and Yuasa, 2005). Interestingly, Nakahira and Yuasa also found that neurons generated at E16 in the mouse CA3 VZ migrate tangentially toward the subpial area, and that some neurons then detach from the stream and migrate radially, with a unipolar shape, along with radial glial fibers directed to the HP of the CA3 region (Nakahira and Yuasa, 2005). This neuronal behavior is different from CA3 neurons generated at E14. Although the CA3 neurons born at E16 account for only a small portion (Bayer, 1980), multiple migration modes may exist for CA3 neurons depending on their birthdates.

The major difference between the CA1 and the CA3 during hippocampal development is the layer shape. The HP in the CA1 is mildly curved and in parallel with its ventricular surface, whereas the CA3 HP has a U-shape with one end invading the dentate hilus. This end is apart from the VZ. Considering that neurons at this end of the HP are also generated from the VZ in the CA3, the long journey for the migrating cells is thought to be one of the causes of delayed HP formation in the CA3, which occurs 1 day later than HP formation in the CA1 (Altman and Bayer, 1990b).

Although the CA1 and the CA3 are continuous architectures via the CA2, pyramidal cells in each region express specific markers; for example, SCIP, a POU domain transcriptional factor, in CA1 pyramidal neurons (Frantz et al., 1994; Tole et al., 1997), and KA1, a glutamate receptor subunit, in CA3 neurons (Wisden and Seeburg, 1993; Tole et al., 1997). Interestingly, these markers are already expressed in each cell group at E15.5 in mice, when the cells are still localized in the IZ (Tole et al., 1997). Explant culture experiments performed by Tole et al. revealed that this marker is expressed in a cell-autonomous manner (Tole and Grove, 2001). Therefore, the destination of cells comprising the HP in the CA3 is thought to already be determined at, or soon after, the multipolar cell stage. For future CA3 pyramidal cells, especially those in the HP end of the dentate hilus, the climbing migration mode may be adequate for migration and detours to reach their final positions. Eventually, CA3 pyramidal neurons might migrate more horizontally through the HP than CA1 pyramidal neurons.

## Migration of cells comprising the dentate gyrus

**Figure 2:**
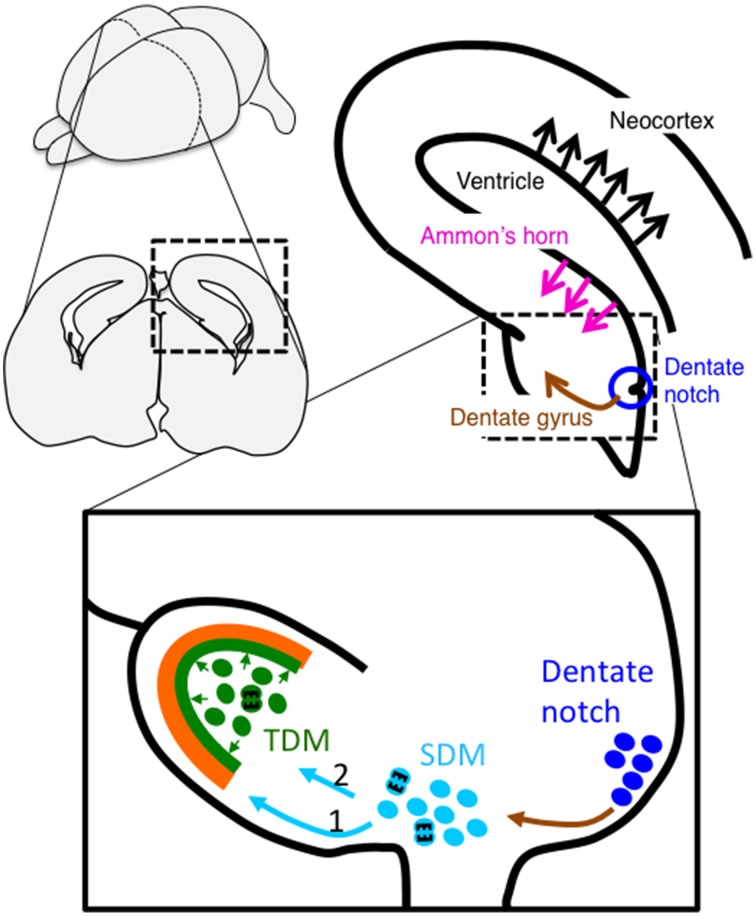
**Schema of migration of dentate cells during hippocampal development**. Newborn granule cells from the dentate notch migrate to the secondary dentate matrix (SDM) (indicated by a brown arrow). The cells then migrate to the subpial surface to form the outer part of the dentate granule cell layer (light blue arrow 1), followed by the dentate hilus (light blue arrow 2), which is called the tertiary dentate matrix (TDM) at this stage, to later form the inner part of the layer. Cells in the TDM exhibit proliferative activities into adulthood, although the proliferative region becomes restricted to the subgranular zone. In contrast, pyramidal neurons in the hippocampal CA1 and neocortex are generated in the Ammonic ventricular zone and the neocortical ventricular zone, respectively, and migrate in a radial direction (indicated by magenta and black arrows, respectively).

Since Altman found postnatal neurogenesis in the subgranular zone (SGZ) of the dentate gyrus (Altman, 1963), it is well-established that the dentate gyrus is one of the two regions where adult neurogenesis occurs. Compared with studies on adult neurogenesis, the development of the dentate gyrus and migratory dynamics of granule cells have been less extensively studied. The generation and migration of dentate cells during development are complex and quite different from pyramidal neurons in the Ammon's horn (Figure 2). Dentate cells are generated in the primary dentate neuroepithelium located around the dentate notch, which is ventral to the Ammonic VZ and dorsal to the fimbria at E16 and E17 of rats (Altman and Bayer, 1990c) and at E13.5 and E14 in mice (Li et al., 2009; Seki et al., 2014). By E15.5 in mice (E18 in rats), some cells that are generated in the primary dentate VZ (Altman and Bayer, 1990c; Seki et al., 2014) migrate out to the subpial region through the suprafimbrial region, which is designated as “dentate migration.” During this migration, these cells still exhibit proliferative activity and form the secondary dentate matrix (SDM in Figure 2) on the migratory route (Altman and Bayer, 1990c; Pleasure et al., 2000). On the migrating stream, the cells extend many processes to various directions (Nakahira and Yuasa, 2005), and the migration route of granule cells and progenitors are separated into two routes. One is the “first dentate migration” in which early-born cells migrate to the crest of the dentate gyrus through the subpial route (route 1 in Figure 2) (Altman and Bayer, 1990c). The dentate cells in the subpial region also exhibit highly proliferative activity (Li et al., 2009). The morphologies of cells in the subpial region are diverse; for example, bipolar morphology and multipolar cell morphology (Nakahira and Yuasa, 2005). This diversity is probably owing to the variety of cellular maturation. The cells reaching the subpial region radially migrate toward the supra-granular blade of the dentate gyrus apposed to the radial glial fibers (Nakahira and Yuasa, 2005). At this stage, these cells form a unipolar or bipolar shape with one or two branches (Nakahira and Yuasa, 2005). The dentate granule cells in the subpial region first form the outer shell of the supra-granular blade of the dentate gyrus, and then gradually shift to form the outer shell of the infra-granular blade of the dentate gyrus. Around E17.5 and 18.5 in mice, a prototype of the dentate gyrus can be observed (Seki et al., 2014). In late embryonic and early postnatal days, the “second dentate migration” appears in which the cells migrate toward and reach the future dentate hilus (route 2 in Figure 2). Because these cells still exhibit proliferative activity even in the hilus, the zone where these cells exist is called the tertiary dentate matrix (TDM in Figure 2). Dentate neurons generated from the TDM form the inner part of the dentate gyrus. As a result, dentate granule cells in the dentate granule cell layer adopt a birthdate-dependent outside-in pattern, in which earlier-born neurons locate to the outer part of this layer and later-born neurons position to the inner region (Rakic and Nowakowski, 1981; Altman and Bayer, 1990c). Although neurogenesis in the TDM continues into adulthood, this zone becomes gradually restricted to the boundary between the dentate granule cell layer and the hilus, called the SGZ (Altman and Bayer, 1990c). Bayer suggests that granule cells in the dentate gyrus are generated from E15 to adulthood in rats, and about 80–85% of total granule cells are generated after birth (Bayer, 1980).

Seki et al. traced the granule cells using a glial fibrillary acidic protein (GFAP)-GFP transgenic mouse line (Seki et al., 2014). GFAP was not thought to be a marker for “embryonic” progenitors of dentate granule cells, although GFAP is a well-known marker for “adult” progenitors. Seki et al. found that migrating progenitors of dentate granule cells, unlike those of neocortical pyramidal neurons, express GFAP from the beginning of dentate development, and these cells could be traced using a GFAP-GFP transgenic mouse line. Immunohistochemical analyses using this transgenic mouse line showed maturation of granule cells during migration. For example, neurogenin-positive proneural cells are mainly localized in the VZ, whereas Tbr2-positive early neural progenitors are principally located in the migratory stream and the developing dentate gyrus. NeuroD-positive immature neurons are mostly located in the migratory stream, the developing dentate gyrus, and the hilus, whereas prox1-positive granule cells are positioned in the developing dentate gyrus and the hilus. Sox2-positive progenitor cells are distributed all over the dentate gyrus at E18 in mice. Accordingly, while these granule cells gradually mature during migration, cells in each region are heterogeneous as to their degree of maturation. How this heterogeneous group could migrate along the same route is not yet known.

## Molecular mechanism of neuronal migration in the hippocampus

A number of mutant mouse lines or the introduction of shRNA-expression vectors for various genes into neurons *in utero* have been used to show abnormal layer formation and mis-positioning of neurons in the Ammon's horn and the dentate gyrus during hippocampal development. Some of the examples are summarized below.

### Reelin (and related molecules, ApoER2, VLDLR, and Dab1)

Reelin is a giant glycoprotein secreted from Cajal–Retzius cells in the MZ during development (D'Arcangelo et al., 1995; Hirotsune et al., 1995; Ogawa et al., 1995). Reelin is known to be essential for neuronal positioning in the brain and spinal cord (Yip et al., 2000, 2011; Honda et al., 2011; Sekine et al., 2014). For example, in the neocortex, the *reelin*-deficient autosomal recessive mouse, *reeler*, displays disrupted layer formation, including overall approximate inversion of the birthdate-dependent layering (Caviness, 1973). Anatomical analyses, such as [3H] thymidine injection and *in situ* hybridization, disclosed that hippocampal layer formation is also inverted in the *reeler* mouse (Caviness, 1973; Stanfield and Cowan, 1979a,b; Stanfield et al., 1979; Niu et al., 2004; Boyle et al., 2011). Injection of CR-50, a function-blocking antibody against Reelin protein, into the ventricle of mouse embryos also resulted in a similar layer pattern to that of *reeler* mice in the hippocampal Ammon's horn (Nakajima et al., 1997). Additionally, *reeler* mice exhibit a divided SP in the CA1, a meandered SP in the CA3, a less densely packed dentate gyrus, and a reduced number of granule cells (Caviness, 1973; Stanfield and Cowan, 1979a,b; Boyle et al., 2011).

Reelin binds to Apolipoprotein E receptor 2 (ApoER2) and very low-density lipoprotein receptor (VLDLR) (D'Arcangelo et al., 1999; Hiesberger et al., 1999; Trommsdorff et al., 1999), subsequently induces phosphorylation of Disabled-1 (Dab1) by Fyn or Src kinases, and then transduces the signal to several downstream pathways to regulate neuronal migration and cellular positioning (Honda et al., 2011; Sekine et al., 2014). Double KO mice of *apoer2* and *vldlr* show similar phenotypes to those of *reeler* mice. Additionally, KO mice of *apoer2* show slightly more severe SP splitting than the *vldlr* KO mice, while the phenotypes of these single KO mice are milder than those of double KO mice (Trommsdorff et al., 1999; Drakew et al., 2002; Weiss et al., 2003). The *dab1* KO mice also exhibit the same hippocampal abnormality as the *reeler* mice (Howell et al., 1997; Weiss et al., 2003).

Analysis of the migratory stream of dentate cells using nestin-GFP transgenic mice mated with *reeler* mice suggests that Reelin is not necessary for the migration of dentate precursors (Nestin-GFP positive cells) from the dentate notch to the subpial zone, but is indispensable for later migration of these cells from the subpial zone to the granule cell layer (Li et al., 2009). Prox1-positive granule cells in these mice are arranged abnormally in the dentate area, suggesting the involvement of Reelin in the final radial migration of dentate granule cells. Forster et al. reported that Reelin and Dab1 affect radial glial cell differentiation and branching in the hippocampus and the loss of these genes causes abnormal radial fiber formation, resulting in failed neuronal migration in the hippocampus (Förster et al., 2002). Zhao et al. rescued *reeler* malformation in the orientation of radial glial fibers and dentate cell migration *in vitro* during dentate gyrus development when they adjacently co-cultured *reeler* dentate gyrus with rat wild-type dentate gyrus (Zhao et al., 2004, 2006). These results collectively imply that Reelin controls neuronal migration in the hippocampus in cell-autonomous and non-cell-autonomous manners.

### Cyclin-dependent kinase 5 (Cdk5)/p35

Cdk5 is a serine/threonine kinase and is activated by binding with its regulatory co-factor p35 or p39. Cdk5 activity is rich in brains during development, and p35 or p39 expressions are restricted to the brain. Cdk5 regulates various aspects of neuronal functions such as cell migration, cytoskeletal remodeling, and adult neurogenesis via phosphorylation of various types of molecules (Su and Tsai, 2011). The *cdk5* KO mice are lethal by birth and display disrupted laminar formation in the neocortex and hippocampus (Ohshima et al., 1996). The *p35* KO mice also exhibit mis-positioning of neuronal cells in the Ammon's horn (partly distinct SP is observed in the CA3, but not in the CA1) and the dentate gyrus, though the phenotypes are moderate compared with *cdk5* KO mice (Wenzel et al., 2001; Ohshima et al., 2005). Cdk5 regulates multipolar-to-bipolar transition of migratory neurons in the neocortex via RapGEF2 phosphorylation (Ohshima et al., 2007; Ye et al., 2014). Because hippocampal pyramidal neurons also perform this transition, a similar mechanism may also play a role during Ammon's horn development.

### Doublecortin (Dcx)

DCX is a microtubule-associated protein involved in neuronal migration and a causative gene for X-linked lissencephaly (des Portes et al., 1998; Gleeson et al., 1998). Patients with lissencephaly with *DCX* mutations exhibit defects in neocortical and hippocampal lamination (Barkovich et al., 1991). Hemizygous male *dcx* mutant mice are lethal by early postnatal days and display disrupted hippocampal formation, that is, abnormal neuronal positioning/migration in the Ammon's horn and partial dividing of the SP in the CA3, while neocortical laminar formation and the dentate gyrus are quite normal (Corbo et al., 2002). The *dcx* heterozygous female mice display milder malformation in the hippocampus and deficits in learning and memory (Corbo et al., 2002). KO mice of *dclk1 or dclk2*, doublecortin-like kinase 1 or 2, respectively, are anatomically normal in the hippocampus (Deuel et al., 2006; Tanaka et al., 2006; Kerjan et al., 2009), but the double KO mice of *dcx* and *dclk1* show severe abnormalities in hippocampal laminar formation in the Ammon's horn and the dentate gyrus (Deuel et al., 2006; Tanaka et al., 2006). In contrast, double KO mice of *dcx* and *dclk2* exhibit disrupted laminar formation in CA1 and CA3 region and a less-packed dentate granule layer (Kerjan et al., 2009). Considering the majority of mutant mice mentioned in this review also show abnormalities in the neocortex, the hippocampus and neocortex are likely to share molecular pathways during development. Mutant mice that exhibit malformations specifically in the hippocampal region, such as *dcx* KO mice, may become a key tool to better understand the molecular mechanisms underlying the unique process of hippocampal development, such as the climbing mode of migration. Analyses of *dcx* KO mice and double KO mice of *dcx* and *dclk1* or *dclk2* may also provide insight into differences between hippocampal CA1 and CA3 regions.

### Pafah1b1 (formerly Lis1)

*PAFAH1B1* is another causative gene for lissencephaly (Reiner et al., 1993; Hattori et al., 1994; Lo Nigro et al., 1997). Pafah1b1 regulates microtubule-based transport by binding with Dynein motor proteins and Ndel1 (formerly Nudel) (Sasaki et al., 2000). Heterozygous *Pafah1b1* KO mice exhibit malformations of the hippocampal cytoarchitecture, which results from delayed neuronal migration and abnormal cellular positioning (Hirotsune et al., 1998; Fleck et al., 2000). Consequently, hippocampal layers in the Ammon's horn become discontinuous and multiple in this mouse, whereas granule cells in the dentate gyrus are less concentrated and loosely packed (Fleck et al., 2000). Another lissencephaly-associated protein, tubulin alpha 1A (Tuba1a), is also involved in hippocampal layer formation (Keays et al., 2007). The *tuba1a* S140G mutant mice induced by injection of N-ethyl-N-nitrosourea (ENU) exhibit deficits in neuronal migration, resulting in a double layer of the hippocampal CA1 and CA3 regions, as well as abnormal laminar formation in the neocortex (Keays et al., 2007).

### Cxcl12 (SDF-1)/Cxcr4

SDF-1 is another secreted protein that regulates cellular migration (Bleul et al., 1996; Ma et al., 1998; Klein et al., 2001). In the dentate gyrus, SDF-1 is expressed in the meninges and Cajal–Retzius cells, whereas its receptor Cxcr4 is expressed in migratory granule cells in the second dentate matrix and the migratory stream (Bagri et al., 2002). Disruption of the normal SDF-1 gradient by the ectopic SDF-1 expression to the hippocampal field using electroporation into slice culture causes a deficit in granule cell migration, suggesting SDF-1 is a chemoattractant factor for dentate migration (Bagri et al., 2002). The *cxcr4* KO mice exhibit a disrupted dentate gyrus, caused by migration defects of granule cells along the subpial stream and subsequent radial migration (Bagri et al., 2002; Lu et al., 2002; Li et al., 2009).

### Nuclear factor Ib (Nfib)

Nfib is a member of nuclear factors I (Nfia, b, c, and d) and functions as a transcriptional factor. The *nfib* KO mice display abnormal hippocampal formation, including the CA3, dentate gyrus, and fimbria, which may be due to aberrant maturation of radial glial fibers in the Ammon's horn (Steele-Perkins et al., 2005; Barry et al., 2008). Although the deficient mouse exhibits normal cell proliferation, the dentate cells accumulate in the subpial region during dentate migration. Barry et al. suggest that this abnormal positioning of dentate cells is attributed to failed radial migration (Barry et al., 2008).

### Disrupted-in-schizophrenia-1 (Disc1)

*DISC1* is a risk gene for major psychiatric disorders, including schizophrenia. We have previously reported that *Disc1* knockdown by *in utero* electroporation causes abnormal migration of hippocampal CA1 neurons; knockdown cells fail to enter the pyramidal layer (Tomita et al., 2011). Disc1 is also related to migration and positioning/integration of dentate granule cells during development and adulthood (Duan et al., 2007; Kim et al., 2009; Meyer and Morris, 2009). KO mice of *girdin*, a Disc1 interacting molecule, show dispersion of granule cells in the dentate gyrus, mis-positioning of pyramidal neurons in the CA1 and the CA3, and split layer in the CA2 (Enomoto et al., 2009). Knockdown of *girdin* in dentate granule cells results in over-migration, as observed for the *Disc1* knockdown cells. Because other genes implicated in neuropsychiatric diseases, such as *CNTNAP2* (Peñagarikano et al., 2011) and *FMRP* (La Fata et al., 2014), are reported to be involved in neuronal migration during neocortical development, it will be interesting to determine whether these genes also affect hippocampal neuronal migration.

Hippocampal neuronal migration is controlled by extracellular factors, such as Reelin, SDF-1, and radial glial fibers, as well as intracellular molecules as mentioned above. Both the *reeler* mouse and the *nfib* KO mouse exhibit abnormal radial glial fibers and disrupted dentate gyrus formation. However, both KO mice exhibit normal dentate cell migration and cells accumulate in the subpial region; the final migration toward the dentate layer is conducted along radial fibers, whereas dentate migration to the subpial region may be independent of these molecules.

Pafah1b1, Dcx, and Tuba1A regulate microtubule dynamics. Dab1 is reported to bind to Lis1 downstream of Reelin signaling (Assadi et al., 2003). Dcx is phosphorylated by Cdk5 at Ser297, resulting in reduced microtubule polymerization and binding affinity to microtubules (Tanaka et al., 2004). Cdk5 also regulates the Pafah1b1-Ndel1-Dynein complex via Ndel1 phosphorylation (Niethammer et al., 2000; Sasaki et al., 2000). Furthermore, Cdk5 and Reelin signaling synergistically contribute to neuronal positioning (Ohshima et al., 2001, 2007; Beffert et al., 2004). Therefore, microtubule dynamics is critical for migration of the hippocampal cells, similar to neocortical neurons.

A number of mutant mice exhibiting abnormal hippocampal formation also display splitting of the SP in the Ammon's horn, although the extent of splitting is not uniform. This may suggest the existence of multiple migration modes for hippocampal pyramidal neurons. In the rodent hippocampus, deep and superficial sublayers are visibly distinguished by cellular density and morphology in the ventral two-thirds of CA1 (Slomianka et al., 2011). Slomianka et al. also reviewed the distinction of histological, molecular, and connective features between deep and superficial sublayers in the hippocampal Ammon's horn (Slomianka et al., 2011). For example, superficial pyramidal cells express Satb2 during development, Nov and Nr3c2 in the hippocampal CA1 in adulthood, and Kcnq5 in the CA3 (Thompson et al., 2008; Dong et al., 2009). In contrast, deep pyramidal neurons express Sox5 during development, Ndst4 and Astn2 in the CA1 in adulthood, and St18 in the CA3 (Thompson et al., 2008; Dong et al., 2009). Furthermore, Mizuseki et al. showed physiological differences between deep and superficial sublayers in the hippocampal CA1 region of rats, such as theta phase preference during REM sleep and gamma phase preference during behavioral task (Mizuseki et al., 2011). In the hippocampal CA1, the climbing mode of migration is observed for late-born neurons (Kitazawa et al., 2014). In contrast, Morest reported that hippocampal cells extended their leading process through the HP and kept it until they reach the pial surface during the early developmental stages of opossum (Morest, 1970). Rodent hippocampal cells may also use this somal-translocation-like mode, especially during early stages of development. If this is the case, early-born neurons and late-born neurons might use different modes of migration and ultimately settle in their respective positions in the SP. The distinct populations between superficial and deep layers may be reflected by specific gene expressions and functions.

## Conclusion

This review discusses the migration of pyramidal neurons in the Ammon's horn and granule cells in the dentate gyrus during hippocampal development. The structure of the hippocampus is dynamically expanded and becomes complicated during development. Because the climbing mode of migration is a flexible migration mode, it may be necessary for hippocampal neurons to accommodate to this hippocampal formation. The migration of dentate cells is well-organized, while cellular maturation is diverse along the migratory stream. Integration of molecular biological studies with histological studies has led to novel discoveries focused on cellular and molecular mechanisms of hippocampal development. Further studies on behaviors of hippocampal neurons are expected in the future to fully understand hippocampal development and functions.

### Conflict of interest statement

The authors declare that the research was conducted in the absence of any commercial or financial relationships that could be construed as a potential conflict of interest.
